# Targeted next-generation sequencing of 491 lung cancers in clinical practice: Implications for future detection strategy and targeted therapy

**DOI:** 10.1016/j.heliyon.2024.e27591

**Published:** 2024-03-07

**Authors:** Xiao-dan Liu, Yan Zhang, Hui-ying He

**Affiliations:** Department of Pathology, School of Basic Medical Sciences, Peking University Third Hospital, Peking University Health Science Center, Beijing, 100191, China

**Keywords:** Non-small cell lung cancer (NSCLC), Next-generation sequencing (NGS), Amplification refractory mutation system PCR (ARMS-PCR), Epidermal growth factor receptor (EGFR), Targeted therapy

## Abstract

Although lung cancer remains the most common cause of global cancer-related mortality, the identiﬁcation of oncogenic driver alterations and the development of targeted drugs has dramatically altered the therapeutic landscape. In this retrospective study, we found that 97.7% samples carried at least one mutation in the 25 genes tested in our cohort. 53.6% samples were positive for EGFR mutations, followed by TP53 (41.1%), KRAS (11.8%), ERBB2 (4.3%). EGFR mutations were mainly found in female adenocarcinomas, while TP53 was mainly found in male non-adenocarcinomas. Significant differences can be found in the mutation rate of EGFR (60.9% vs 11.9%), KRAS (12.2% vs 25.0%), STK11 (1.5% vs 11.9%), FGFR3 (2.4% vs 0.0%) and ERBB4 (1.2% vs 6.1%) between adenocarcinoma in our cohort and TCGA-LUAD data (all p < 0.001). What's more, we found that the mutation of EGFR increased significantly from adenocarcinomas in situ (AIS, 21.4%) to microinvasive adenocarcinomas (MIA, 52.4%) and invasive adenocarcinomas (IA, 61.1%), while the mutation of ERBB2 dropped markedly from AIS (21.4%) to MIA (9.5%) and IA (4.1%). At last, comparations between targeted NGS and ARMS-based single gene test in the detection of EGFR showed a 94.6% consistence. In conclusion, targeted NGS can provide a comprehensive mutational profile of lung cancer. Considering the high mutation rate of EGFR in NSCLC of Asian populations, a specialized detection strategy should be conducted.

## 1 Introduction

Lung cancer is the most common cause of global cancer-related mortality, leading to over a million deaths each year, and more than 85% of those cases are currently classified as non-small cell lung cancer (NSCLC) [1]. Technological advances during the past decade, including the introduction of next-generation sequencing (NGS) and the construction of large databases characterizing the molecular features of human tumors, have transformed our view of NSCLC from histopathological descriptions to precise molecular and genetic identities. Recently, molecularly targeted therapies have dramatically improved treatment for patients whose tumors harbor somatically activated oncogenes such as mutant EGFR [2] or translocated ALK, RET, or ROS1 [[3], [4], [5]]. The National Comprehensive Cancer Network treatment guidelines advocate actionable mutation screening as standard of care [6].

As the number of emerging biomarkers and targets continues to grow, single EGFR gene test or multiplexed gene test covering most of the targetable genes (EGFR, ALK, ROS1, RET, BRAF) as we used previously couldn't fulfill the need of clinics. In contrast, comprehensive next-generation sequencing (NGS), such as Whole Genome Sequencing (WGS) or Whole Exome Sequencing (WES) can sequence all the genes or exons simultaneously, with the limitations of high cost and long turn-around time. Compared to single gene assay or WGS/WES, targeted next-generation sequencing (NGS) is a cost- and time-effective platform to detect multiple mutations simultaneously in various genes with high reproducibility and sensitivity [7]. Thus, targeted NGS was introduced in our routine molecular test, and the mutation profile of Chinese lung cancer patients tested in our department of pathology was retrospectively analyzed. Giant differences in the mutation profile were observed between Chinese and Western lung cancer patients. Comparations of targeted NGS panel with allele specific real-time PCR in the detection of EGFR showed a 94.6% consistence. Our study will advance our understanding of the molecular profile of NSCLC, and provides important information for targeted therapy in clinic.

## 2 Materials and methods

### 2.1 Patients and samples

A total of 491 samples from 480 lung cancer patients were addressed to our laboratory for molecular diagnosis from March 2020 to October 2021. The histological classifications of all samples were reviewed independently by two pathologists, according to the lung cancer classiﬁcation of the International Association for the Study of Lung Cancer, American Thoracic Society [8]. For samples with tumor cells <20%, microdissection was performed. This study was approved by Peking University Third Hospital Medical Science Research Ethics Committee (approval number: S2023761). Informed consents of all patients were obtained from patients themselves or their relatives.

### 2.2 DNA and RNA extraction

Formalin-Fixed Paraffin-Embedded (FFPE) cancer samples were used for the DNA and RNA extraction, which was performed using a AllPrep DNA/RNA FFPE Kit (Cat^#^ 80234, Qiagen, Germany), following the manufacturer's instructions. The optical density of the extracted DNA/RNA samples was measured using a Nanodrop 2000 or Qubit 3 (Thermo fisher, USA). The A260/A280 value of all the samples was 1.8–2.1.

### 2.3 Targeted next-generation sequencing

NGS was performed to sequence more than 1600 hotspot mutations in AKT1, ALK, BRAF, CTNNB1, DDR2, EGFR, ERBB2, ERBB4, FBXW7, FGFR1, FGFR2, FGFR3, KRAS, MAP2K1, MET, NOTCH1, NRAS, NTRK1, PIK3CA, PTEN, RET, ROS1, SMAD4, STK11 and TP53 as well as UGT1A1 rs4148323 polymorphism (Supplemental Table 1) (human EGFR/KRAS/BRAF/PIK3CA/ALK/ROS1 kit, Cat^#^ CP-001; Novogene, China). 10 ng DNA and RNA per sample was used for library preparations according to manufacturer's instructions. The amplicon-based NGS panel were sequenced on the Proton DA8600. DNA-based data test for SNVs and InDels, and RNA-based data test for gene fusions. For DNA analysis, align raw data to hg19 using BWA [9], Vardict-java [10] and Mutect2 [11] were used to detect somatic mutations. Only the somatic mutations with frequency≥0.5% and both called out by Vardict-java and Mutect2 were kept in the analysis. For RNA analysis, alignment and fusion calling were performed by STAR-Fusion [12]. For data QC, bamdst (https://github.com/shiquan/bamdst) and samtools [13] were used, a sample's QC passed with average sequencing depth ≥1000X, uniformity ≥90%, mapping rate ≥95%, on-target rate ≥90%, and the RNA reads number >60,000.

### 2.4 Amplification refractory mutation system (ARMS) method

The EGFR mutation status of most of the samples was also validated with the ARMS method in parallel with the NGS test, according to the manufacturer's instructions (human EGFR test kit, Cat^#^ 8.01.0130, Amoy Diagnostics Ltd, China). An 8-tube strip for PCR, with 10 ng DNA added into each tube, was used for the amplification of EGFR gene. Each tube was pre-loaded with the mixture of primers and Taqman probes, which can be used for the detection of different hotspot mutations. The detailed exons and covered sites can be found in Supplemental Table 2. The ARMS assay was conducted on the Mx3000P system (Agilent Technologies, USA), with PCR amplification protocol as follows: Stage 1: 95 °C, 5 min, 1 cycle; Stage 2: 15 cycles of (95 °C, 25 s; 64 °C, 20 s; 72 °C, 20 s); Stage 3: 31 cycles of (93 °C, 25 s; 60 °C, 35 s; 72 °C, 20 s). Fluorescence was collected at 60°Cof stage 3. The detection sensitivity of ARMS was 1%.

### 2.5 Analysis of LUAD data from the cancer Genome Atlas (TCGA) database

We used the RStudio software (Version 1.3.1093) for bioinformatic analysis. Masked Somatic Mutation files of 569 cases of LUAD was downloaded from the TCGA database (https://portal.gdc.cancer.gov/). Mutation frequencies of the same 25 genes as we examined in our NGS panel was analyzed with the “Maftools” package, and Waterfall plot was drawn using the “oncostrip” package.

### 2.6 Statistical analysis

Statistical analysis was conducted by using GraphPad Prism software (version 8, GraphPad Software, USA). The Chi-square, Fisher exact, and Kruskal-Wallis tests were used to calculate the signiﬁcance of the differences between different subsets. All reported p values less than 0.05 were deﬁned as signiﬁcantly different.

## 3 Results

### 3.1 Demographic and clinicopathological data of the patients

The demographics of 491 lung cancer patients enrolled consecutively from March 2020 to October 2021 in our molecular lab (Defined as ‘PUTH cohort’ hereafter) are summarized in Table 1. Male and female patients are almost equal in number (50.5% vs 49.5%). The age range of patients at diagnosis was 24–93 years (median: 65 years), of which 3.7% patients under 40 years, 33.8% patients at 41–60 years, 57.4% patients at 61–80 years and 5.1% patients over 81 years. Regarding the histological subtypes, all tumor samples were classified as Adenocarcinoma (83.3%), Squamous cell carcinoma (7.5%), Adenosquamous carcinoma (1.8%), Sarcomatoid carcinoma (1.4%), Small cell carcinoma (1.0%), NSCLC-NOS (2.0%), as well as Precursor glandular lesions (2.9%). Specimens were sampled by resection (52.5%), core needle lung biopsy (29.5%), bronchial biopsy (9.8%) or pleural effusion exfoliated cells (8.1%).

**Table 1:** Demographics of the 491 patients with lung cancer.

		No. of cases	Percentage (%)
Sex	Male	248	50.5%
	Female	243	49.5%
Age(y)	Range (24–93), Median (65)		
	20–40	18	3.7%
	41–60	166	33.8%
	61–80	282	57.4%
	>81	25	5.1%
Pathological types	Adenocarcinoma	409	83.3%
	Squamous cell carcinoma	37	7.5%
	Adenosquamous carcinoma	9	1.8%
	Sarcomatoid carcinoma	7	1.4%
	Small cell carcinoma	5	1.0%
	NSCLC-NOS	10	2.0%
	Precursor glandular lesions	14	2.9%
Specimen	Resected tissue	258	52.5%
	Punctured lung tissue	145	29.5%
	Bronchial biopsied tissue	48	9.8%
	Pleural effusion exfoliated cells	40	8.1%

### 3.2 Mutational profile of PUTH cohort

**Fig. 1:**
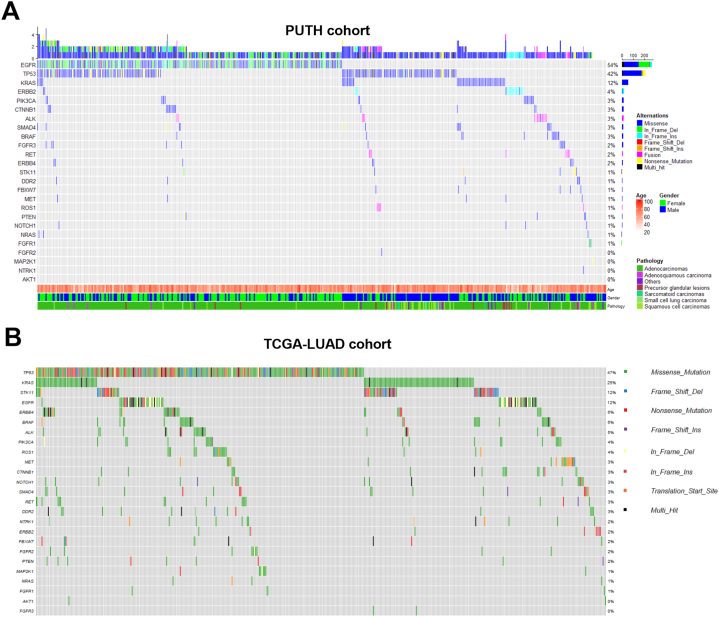
Mutational landscape of the PUTH cohort and TCGA-LUAD cohort (A) Comprehensive visualized plot of the PUTH cohort consisting of 491 lung cancer patients. Top bar graph describes the mutation number in each patient. The mutational frequencies of individual genes were shown on the right. Key characteristics, including age, sex, and pathological types, are presented as a heatmap below the plot. (B) Visualized plot of TCGA-LUAD cohort.

In the 491 samples tested with targeted NGS, 97.7% samples carried at least one mutation in the 25 genes. 53.8% samples were positive for EGFR mutations, followed by TP53 (41.1%), KRAS (11.8%), ERBB2 (4.3%), [PIK3CA, CTNNB1, ALK, SMAD4, BRAF] (∼3%), [FGFR3, RET, ERBB4] (∼2%), while mutations in AKT1, DDR2, FBXW7, FGFR1, FGFR2, MAP2K1, MET, NOTCH1, NRAS, NTRK1, PTEN, ROS1, STK11 were rare (1% or less) (Fig. 1A).

We then correlated the top 3 mutated genes (EGFR, TP53 and KRAS) with clinicopathological data. None of the mutational status of the three genes was correlated with age when the cohort was divided into <65 or ≥65 groups (p > 0.05). Instead, the mutation rate of EGFR was significantly higher in female (69.96%) than in male (37.90%) patients (p < 0.0001), while the mutation rate of TP53 in male (50.40%) was much higher than in female (31.70%) patients (p < 0.0001). In contrast, no difference was found in the mutation rate of KRAS between male and female (p > 0.05). Considering the pathological types of lung cancers, EGFR mutations were mainly found in adenocarcinomas compared to non-adenocarcinomas (61.12% vs 17.07%, p < 0.0001), while mutations in TP53 were mainly found in non-adenocarcinomas compared to adenocarcinomas (53.70% vs 38.60%, p < 0.05). No significant difference was found in the mutation of KRAS between adenocarcinomas and non-adenocarcinomas (p > 0.05). We then examined whether the sampling method (resected tissue, biopsied tissue or pleural effusion exfoliated cells) has influence on the mutation rate of EGFR, TP53 and KRAS. No significant difference was found among the three methods in the detection of EGFR and KRAS (p > 0.05), but the mutation rate of TP53 in biopsied tissue was much higher than the other two methods (p < 0.001). (Table 2).

**Table 2:** Correlation analysis of the mutation rates of the common genes with clinical data.

		EGFR	TP53	KRAS
Cases (%)		264 (53.77%)	202 (41.11%)	58 (11.81%)
Age (y)	<65	62 (55.85%)	49 (44.10%)	14 (12.61%)
	≥65	202 (53.16%)	153 (40.30%)	44 (11.58%)
	Chi-square	0.2516	0.5343	0.0881
	P value	>0.05	>0.05	>0.05
Sex	Male	94 (37.90%)	125 (50.40%)	35 (14.11%)
	Female	170 (69.96%)	77 (31.70%)	23 (9.47%)
	Chi-square	50.74	17.75	2.545
	P value	<0.0001	<0.0001	>0.05
Pathological types	Adenocarcinomas	250 (61.12%)	158 (38.60%)	50 (12.22%)
	Non-Ac	14 (17.07%)	44 (53.70%)	8 (9.76%)
	Chi-square	53.32	6.37	0.40
	P value	<0.0001	<0.05	>0.05
Specimen	Resected tissue	137 (53.10%)	86 (33.33%)	27 (10.46%)
	Biopsied tissue	101 (52.33%)	99 (51.29%)	26 (13.47%)
	Pleural effusion exfoliated cells	26 (65.00%)	17 (42.5%)	5 (12.50%)
	Chi-square	2.236	14.72	0.98
	P value	>0.05	<0.001	>0.05

### 3.3 Mutational profile of TCGA-LUAD cohort

We also analyzed the mutational profile of the LUAD data from TCGA database, where the samples were almost all adenocarcinomas and detected by whole exome sequencing. We extracted the data of the same 25 genes as tested in the PUTH cohort for comparations. TP53 (47.1%), KRAS (25.0%), EGFR (11.9%), STK11(11.9%) were among the top 4 altered genes, followed by [ERBB4, BRAF] (∼6%), ALK (5.0%), [PIK3CA, ROS1] (∼4%), [MET, CTNNB1, NOTCH1, SMAD4, RET, DDR2] (∼3%), [NTRK1, ERBB2, FBXW7, FGFR2, PTEN] (∼2%), while mutations in MAP2K1, NRAS, FGFR1, AKT1, FGFR3 were rare (∼1% or less) (Fig. 1B).

We then compared the mutation rate of adenocarcinomas in the PUTH cohort (n = 409) with TCGA-LUAD data. Significant differences can be found in the mutation rate of EGFR (60.9% in PUTH cohort vs 11.9% in TCGA-LUAD, p < 0.0001), KRAS (12.2% vs 25.0%, p < 0.0001), STK11 (1.5% vs 11.9%, p < 0.0001), FGFR3 (2.4% vs 0.0%, p < 0.001) and ERBB4 (1.2% vs 6.1%, p < 0.001). Meanwhile, mutation rate of TP53 (37.7 vs 47.1%, p < 0.01) and 7 other genes (BRAF, DDR2, ERBB2, FGFR2, NOTCH1, NTRK1, ROS1) showed a slight but statistically significant difference (all p < 0.05, data not shown). In contrast, no difference was found in the mutation rate of the rest 12 genes (Table 3).

**Table 3:** Comparations in the mutation rate of different genes in adenocarcinoma between PUTH and TCGA cohort.

Cohort	Cases	EGFR	TP53	KRAS	FGFR3	STK11	ERBB4
PUTH	409	60.9%	37.7%	12.2%	2.4%	1.5%	1.2%
TCGA	561	11.9%	47.1%	25.0%	0.0%	11.9%	6.1%
Chi-square		257.9	8.534	24.34	13.86	37.30	14.35
P value		<0.0001	<0.01	<0.0001	<0.001	<0.0001	<0.001

#### 3.3.1 Mutation profile of driver genes in AIS, MIA and IA

Although several studies have identiﬁed mutations shared by lung cancer drivers in AIS and MIA [[14], [15], [16], [17], [18]], the mutation rate of the three key driver genes (TP53, EGFR and KRAS) varied among these studies, so we compared the mutation profile of these genes in our cohort. Interestingly, we found that the mutation of EGFR increased significantly from AIS (21.4%) to MIA (52.4%) and IA (61.1%), while the mutation of ERBB2 dropped markedly from AIS (21.4%) to MIA (9.5%) and IA (4.1%). Instead, no significant difference was found in the mutation rate of TP53 among the three groups (Table 4).

**Table 4:** Comparations in the mutation rate of the common genes among AIS, MIA and IA.

Cohort	Cases	EGFR	TP53	KRAS	ERBB2
AIS	14	21.4%	21.4%	21.4%	21.4%
MIA	21	52.4 %	23.8%	0.0%	9.5%
IA	388	61.1 %	37.9%	12.9%	4.1 %
Chi-square		9.249	3.144	4.065	9.550
P value		<0.01	>0.05	>0.05	<0.01

Due to the small number of cases of AIS and MIA, we reviewed the molecular test results of AIS and MIA in the past five years in our hospital to confirm the result. Collectively, there were 51 and 43 cases of AIS or MIA with EGFR test, separately. Mutation of EGFR were found in 23.5% of AIS and 58.1% of MIA samples (Table 5), which was consistent with the result above. We have no mutation data of ERBB2 to review as it was not routinely tested in practice.

**Table 5:** Comparations in mutation rate of EGFR among AIS, MIA and IA.

Cohort	Cases	EGFR
AIS	51	23.5%
MIA	43	58.14%
IA	388	61.1 %
Chi-square		25.94
P value		<0.0001

### 3.4 Comparations between targeted NGS and single gene test

**Fig. 2:**
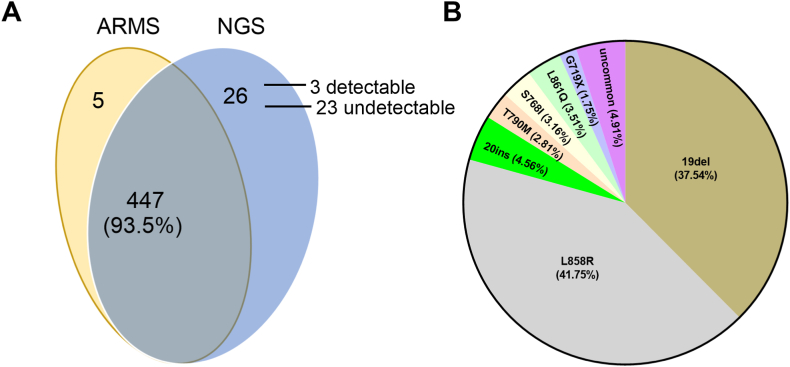
Comparations between NGS and ARMS in the detection of EGFR. (A) Venn diagram shows the consistence between NGS and ARMS in the detection of EGFR. (B) Pie chart shows all the mutational pattern in EGFR detected in our cohort.

In PUTH cohort, 478 samples (97.4%) were also tested with single EGFR test using the ARMS assay in parallel. We analyzed the concordance of both assays in the detection of EGFR. 452 cases (94.6%) showed the same result, including 238 cases were tested positive and 214 cases negative by both assays. Among the 26 inconsistent cases, 22 samples were tested positive only by NGS. When reviewing the mutation sites detected, 19 cases carried uncommon mutations that were not covered by the ARMS assay, while only 3 cases carried hotspot mutations that could be detected by the ARMS assay (false negative). The rest 4 samples were tested positive only by the ARMS assay (Fig. 2A, Supplemental Table 3). The discordance in mutations of these 7 samples between ARMS and NGS was possibly due to the insufficient tumor cells in either method, as all of the 7 samples were biopsied tissue or pleural effusion exfoliated cells.

In general, 285 mutations in EGFR were detected in the 264 mutant samples. L8585R, exon 19 deletions (E19dels) accounted for 41.7%, 37.5% of all mutations, separately (Fig. 2B). 244 samples carried one single mutation, 18 carried two mutations and 2 samples carried three mutations. In the 8 cases harboring T790 M mutations, 6 cases co-aggregated with E19dels, 2 cases co-aggregated with L858R; In the 9 cases of S768I mutations detected, 5 co-aggregated with G719X, 3 co-aggregated with L858R, and 1 co-aggregated with G724S. This suggested that T790 M and S768I all exist combined with other mutations.

### 3.5 Targeted therapy associated gene mutations

**EGFR** In the 264 patients with EGFR mutations, 212 patients harboring single L858R or E19dels could benefit from first (Erlotinib, Gefitinib) or second-generation (Afatinib and Dacomitinib) tyrosine kinase inhibitors (TKIs) [19]; 19 patients with G719X/S768I/L861Q mutations could benefit from Afatinib treatment [20]; 13 patients with exon 20 insertions can be treated with Amivantamab [21] or Mobocertinib [22]. 7 patients with L858R/E19dels + T790 M mutation had a history of 1st generation TKIs treatment and subsequent tolerance, and has changed to Osimertinib treatment thereafter [23]. 2 patients with E19dels + T790 M + C797S mutations had a sequential treatment of 1st and 3rd TKIs, and now turned to 1st TKIs treatment again [24].

**ALK, ROS1 and RET arrangement** 14 ALK and 4 ROS1 rearrangements were detected in our cohort, patients with those mutations can benefit from Crizotinib, Ceritinib, Lorlatinib or Entrectinib treatment [19]. In addition, 8 patients with RET rearrangement can be treated with Selpercatinib [25] or Pralsetinib [26], the detailed rearrangement type can be found in the Table 6.

**Table 6:** Gene mutation status and the respective targetable drugs.

Gene	Mutation	Cases	Drug
EGFR	Ex19 dels	101	Erlotinib, Gefitinib
	L858R	111	
	Ex19 dels + T790 M + C797S	2	
	L861Q	10	Afatinib
	S768I + G719X/L858R/G724S	9	
	Ex20 insertions	13	Amivantamab, Mobocertinib
	Ex19 dels/L858R + T790 M	7	Osimertinib
ALK	EML4 (E6)-ALK (E20)	6	Crizotinib, Ceritinib,Alectinib, Brigatinib,Lorlatinib
	EML4 (E20)-ALK (E20)	5	
	EML4 (E13)-ALK (E20)	3	
ROS1	SDC4(E2)-ROS1(E32)	2	Crizotinib, Ceritinib,Lorlatinib, Entrectinib
	SDC4(E4)-ROS1(E32)	1	
	CD74(E6)-ROS1(E34)	1	
RET	KIF5B(E15)-RET(E12)	6	Selpercatinib, Pralsetinib
	KIF5B(E16)-RET(E12)	1	
	CCDC6(E1)-RET(E12)	1	
BRAF	V600E	5	Dabrafenib, Trametinib
ERBB2	Ex20 insertions	14	Trastuzumab, Deruxtecan
KRAS	G12C	19	Sotorasib, Adagrasib

**BRAF** 13 BRAF gene mutations were detected in our cohort, among which 5 were V600E mutation, which can be treated with the combination of Dabrafenib and Trametinib [27].

**HER2** Trastuzumab deruxtecan is an antibody-conjugated drug targeting HER2 exon 20 insertions, which demonstrated an objective response rates (ORR) of 55% and a median progression-free survival (PFS) of 8.2 months in the phase II DESTINY-Lung01 trial [28]. 14 out of the 21 mutations in HER2 detected in our cohort were exon 20 insertions, which suggest these patients can be treated with Trastuzumab deruxtecan.

**KRAS** KRAS ranked the top 3 mutated genes in our cohort, totally 58 mutations in KRAS were detected, including 19 G12C, 15 G12D, 11 G12V and 13 other mutations. KRAS mutations were not druggable for a long time, until the approval of Sotorasib by U.S. Food and Drug Administration (FDA) for the treatment of KRAS G12C mutations [29]. Those patients with KRAS G12C mutations can also be treated with Adagrasib, which demonstrated an ORR of 45% in the KRYSTAL-1 phase I and II trial [30] and has received FDA breakthrough therapy designation.

## 4 Discussion

Although lung cancer remains the most commonly diagnosed cancer and the leading cause of cancer death globally [1], the identiﬁcation of oncogenic driver alterations by NGS and development of targeted drugs has dramatically altered the therapeutic landscape. Here, we reviewed the mutation data of lung cancer by NGS, from March 2020 to October 2021 in our hospital and examined the efficacy of NGS in molecular diagnosis and the guidance for targeted therapy.

In general, 97.7% samples carried at least one mutation in one of the 25 genes, showing a wide coverage of this gene panel for lung cancer. In adenocarcinomas, EGFR (60.9%), TP53 (37.7%) and KRAS (12.2%) were the top 3 frequently mutated genes in Chinese people, which is significantly different from Western people, as compared with the TCGA-LUAD data (TP53–47.1%, KRAS-25.0%, EGFR-11.9%). This suggested significant geographical diversity for EGFR mutations between Asian and Western NSCLC populations, which is consistent with previous report [31,32]. A recent study explored the genomic landscape of lung adenocarcinomas in different races, and found significant difference in the mutation rate of clinical actional mutations among different ethnicities. For example, the EGFR L858R mutation was three times higher in Asians than in all other races. White patients had the highest rate of KRAS G12C (15.51%) alteration than other races (P < 0.001). ALK rearrangement, RET rearrangement and ERBB2 ampliﬁcation were more common in Asian patients than in Other racial groups [33]. We summarized in Table 6 the targetable genes and respective drugs in our cohort. Collectively, 64.6% (317/491) patients harbored mutations that were targetable, in which 79.8% (253/317) patients were those harboring EGFR mutations. Meanwhile, assay comparations between single EGFR test based on ARMS-PCR and targeted NGS showed that 94.6% cases were consistent. Due to the giant difference in mutation profile between Asian and Western NSCLC populations, the choice of gene panel and detection strategies for the targeted treatment of NSCLC should be specialized made for Asian populations. For example, a guideline made by Chinese Society of Pathology et al. recommended multiplexed PCR test including necessary genes (EGFR, ALK, ROS1 and MET-exon 14 skipping) and expanded genes (MET amplification, HER2, BRAF, RET, KRAS, NTRK) for the molecular tests in NSCLC as a priority [34]. Further studies are required to compare multiplexed PCR test with targeted NGS in the detection of targetable driver genes of NSCLC.

Correlations with the top 3 mutated genes (EGFR, TP53, KRAS) with patients’ demographics showed that EGFR was mainly found in female, adenocarcinomas, while TP53 was mainly found in male, non-adenocarcinomas. No significant difference was found in the mutation of these 3 genes among different sampling method (resected tissue, biopsied tissue or pleural effusion exfoliated cells), except for TP53, which showed a slightly higher mutation rate in biopsied tissue than sampling by another two methods (p < 0.001). This is possibly because of a higher rate of non-adenocarcinomas sampled by biopsy (42/193, 21.76%) than cytology (1/40, 2.5%) or resection (39/258, 15.12%) in our cohort. Tumor heterogeneity could also lead to this discrepancy by different sampling method. To avoid this, liquid biopsy can serve as a complement [35]. But under the premise of tumor tissue available, the genetic testing using tissue is preferred.

Exploring the key molecules driving the lung tissue from non-invasive (atypical adenomatous hyperplasia) to pre-invasive (AIS and MIA) and fully invasive adenocarcinomas is critical to elucidate the mechanism of early invasive progression, classify molecular genotypes, and provide potential strategies for early intervention. However, findings from previous studies on the early invasive events were conflicting [14,15,18,[36], [37], [38], [39]]. For example, results from Evgeny and Wang et al. showed KRAS, TP53, and EGFR mutations played a dominant role in early invasive LUAD [18,36],while Zhang et al. highlighted the role of EGFR, ERBB2, and BRAF as early clonal genomic events in AIS, but TP53 was mainly found in MIA and IA [14]. These discrepancies are possibly due to the small number of cases enrolled for AIS and MIA in each study. Our NGS result showed that EGFR mutations increased from AIS (21.4%) to MIA (52.4%) and IA (61.1%), while the mutation of ERBB2 dropped markedly from AIS (21.4%) to MIA (9.5%) and IA (4.1%). This is consistent with a recently published study based on the largest cohort by present, in which they found a high mutation rate of EGFR (30.1%), ERBB2 (23.2%), BRAF (16.7%), and MAP2K1 (10.6%) in AIS. Except for EGFR, mutation rate of ERBB2, BRAF, and MAP2K1 were all inversely proportional with the general invasion pattern, with the highest rates found in AIS followed by MIA, and the lowest rates found in IA [40]. All these studies highlighted the role of EGFR in the transition of lung cancer from preinvasive to invasive status.

As a conclusion, our study advances the understanding of the molecular profiles of lung cancer, and most importantly provides invaluable information for targeted therapy in clinic. Considering the high mutation rate of EGFR in NSCLC of Asian populations, as well as the concordance in the detection of EGFR between ARMS-based single gene test and NGS, the optimal detection strategies should be specialized made for Asian populations, especially in less developed area, more economic and faster ARMS assay could be a priority selection.

## Ethics approval

This study was approved by Peking University Third Hospital Medical Science Research Ethics Committee (approval number: S2023761). Informed consents of all patients were obtained from patients themselves or their relatives. The authors declare no competing interests.

## Data availability statement

All data associated with this study was included in the article/supplemental materials.

## CRediT authorship contribution statement

**Xiao-dan Liu:** Writing – review & editing, Writing – original draft, Methodology, Investigation, Formal analysis, Data curation, Conceptualization. **Yan Zhang:** Visualization, Investigation. **Hui-ying He:** Writing – review & editing, Validation, Supervision, Conceptualization.

## Declaration of competing interest

The authors declare no competing interests.

## References

[1] Siegel R.L., Miller K.D., Fuchs H.E. (2021). Cancer Statistics, 2021. CA: a cancer journal for clinicians.

[2] Paez J.G., Janne P.A., Lee J.C. (2004). EGFR mutations in lung cancer: correlation with clinical response to gefitinib therapy. Science (New York, N.Y.).

[3] Bergethon K., Shaw A.T., Ou S.H. (2012). ROS1 rearrangements define a unique molecular class of lung cancers. J. Clin. Oncol. : official journal of the American Society of Clinical Oncology.

[4] Drilon A., Wang L., Hasanovic A. (2013). Response to Cabozantinib in patients with RET fusion-positive lung adenocarcinomas. Cancer Discov..

[5] Kwak E.L., Bang Y.J., Camidge D.R. (2010). Anaplastic lymphoma kinase inhibition in non-small-cell lung cancer. N. Engl. J. Med..

[6] Ettinger D.S., Wood D.E., Aisner D.L. (2017). Non-small cell lung cancer, version 5.2017 clinical practice guidelines in oncology. J. Natl. Compr. Cancer Netw..

[7] Legras A., Barritault M., Tallet A. (2018). Validity of targeted next-generation sequencing in routine care for identifying clinically relevant molecular profiles in non-small-cell lung cancer: results of a 2-year experience on 1343 samples. J. Mol. Diagn. : J. Mod. Dynam..

[8] Travis W.D., Brambilla E., Nicholson A.G. (2015). The 2015 world health organization classification of lung tumors: impact of genetic, clinical and radiologic advances since the 2004 classification. J. Thorac. Oncol. : official publication of the International Association for the Study of Lung Cancer.

[9] Li H., Durbin R. (2010). Fast and accurate long-read alignment with Burrows-Wheeler transform. Bioinformatics.

[10] Lai Z., Markovets A., Ahdesmaki M. (2016). VarDict: a novel and versatile variant caller for next-generation sequencing in cancer research. Nucleic Acids Res..

[11] Cibulskis K., Lawrence M.S., Carter S.L. (2013). Sensitive detection of somatic point mutations in impure and heterogeneous cancer samples. Nat. Biotechnol..

[12] Haas B.J., Dobin A., Li B. (2019). Accuracy assessment of fusion transcript detection via read-mapping and de novo fusion transcript assembly-based methods. Genome Biol..

[13] Li H., Handsaker B., Wysoker A. (2009). The sequence alignment/Map format and SAMtools. Bioinformatics.

[14] Zhang C., Zhang J., Xu F.P. (2019). Genomic landscape and immune microenvironment features of preinvasive and early invasive lung adenocarcinoma. J. Thorac. Oncol. : official publication of the International Association for the Study of Lung Cancer.

[15] Chen H., Carrot-Zhang J., Zhao Y. (2019). Genomic and immune profiling of pre-invasive lung adenocarcinoma. Nat. Commun..

[16] Vinayanuwattikun C., Le Calvez-Kelm F., Abedi-Ardekani B. (2016). Elucidating genomic characteristics of lung cancer progression from in situ to invasive adenocarcinoma. Sci. Rep..

[17] Kobayashi Y., Mitsudomi T., Sakao Y. (2015). Genetic features of pulmonary adenocarcinoma presenting with ground-glass nodules: the differences between nodules with and without growth. Ann. Oncol. : official journal of the European Society for Medical Oncology.

[18] Izumchenko E., Chang X., Brait M. (2015). Targeted sequencing reveals clonal genetic changes in the progression of early lung neoplasms and paired circulating DNA. Nat. Commun..

[19] Tan A.C., Tan D.S.W. (2022). Targeted therapies for lung cancer patients with oncogenic driver molecular alterations. J. Clin. Oncol. : official journal of the American Society of Clinical Oncology.

[20] Yang J.C.-H., Schuler M., Popat S. (2020). Afatinib for the treatment of NSCLC harboring uncommon EGFR mutations: a database of 693 cases. J. Thorac. Oncol..

[21] Park K., Haura E.B., Leighl N.B. (2021). Amivantamab in EGFR exon 20 insertion-mutated non-small-cell lung cancer progressing on platinum chemotherapy: initial results from the CHRYSALIS phase I study. J. Clin. Oncol. : official journal of the American Society of Clinical Oncology.

[22] Riely G.J., Neal J.W., Camidge D.R. (2021). Activity and safety of Mobocertinib (TAK-788) in previously treated non-small cell lung cancer with EGFR exon 20 insertion mutations from a phase I/II trial. Cancer Discov..

[23] Mok T.S., Wu Y.L., Ahn M.J. (2017). Osimertinib or Platinum-Pemetrexed in EGFR T790M-positive lung cancer. N. Engl. J. Med..

[24] Leonetti A., Sharma S., Minari R. (2019). Resistance mechanisms to osimertinib in EGFR-mutated non-small cell lung cancer. Br. J. Cancer.

[25] Drilon A., Oxnard G.R., Tan D.S.W. (2020). Efficacy of Selpercatinib in RET fusion-positive non-small-cell lung cancer. N. Engl. J. Med..

[26] Griesinger F., Curigliano G., Thomas M. (2022). Safety and efficacy of pralsetinib in RET fusion-positive non-small-cell lung cancer including as first-line therapy: update from the ARROW trial. Ann. Oncol. : official journal of the European Society for Medical Oncology.

[27] Planchard D., Smit E.F., Groen H.J.M. (2017). Dabrafenib plus trametinib in patients with previously untreated BRAF(V600E)-mutant metastatic non-small-cell lung cancer: an open-label, phase 2 trial. Lancet Oncol..

[28] Li B.T., Smit E.F., Goto Y. (2022). Trastuzumab deruxtecan in HER2-mutant non-small-cell lung cancer. N. Engl. J. Med..

[29] Skoulidis F., Li B.T., Dy G.K. (2021). Sotorasib for lung cancers with KRAS p.G12C mutation. N. Engl. J. Med..

[30] Janne P.A., Riely G.J., Gadgeel S.M. (2022). Adagrasib in non-small-cell lung cancer harboring a KRAS(G12C) mutation. N. Engl. J. Med..

[31] Chen J., Yang H., Teo A.S.M. (2020). Genomic landscape of lung adenocarcinoma in East Asians. Nat. Genet..

[32] Tan D.S., Mok T.S., Rebbeck T.R. (2016). Cancer genomics: diversity and disparity across ethnicity and geography. J. Clin. Oncol. : official journal of the American Society of Clinical Oncology.

[33] Shi H., Seegobin K., Heng F. (2022). Genomic landscape of lung adenocarcinomas in different races. Front. Oncol..

[34] (2021).

[35] Canale M., Pasini L., Bronte G. (2019). Role of liquid biopsy in oncogene-addicted non-small cell lung cancer. Transl. Lung Cancer Res..

[36] Wang S., Du M., Zhang J. (2020). Tumor evolutionary trajectories during the acquisition of invasiveness in early stage lung adenocarcinoma. Nat. Commun..

[37] Qian J., Zhao S., Zou Y. (2020). Genomic underpinnings of tumor behavior in in situ and early lung adenocarcinoma. Am. J. Respir. Crit. Care Med..

[38] Sivakumar S., Lucas F.A.S., McDowell T.L. (2017). Genomic landscape of atypical adenomatous hyperplasia reveals divergent modes to lung adenocarcinoma. Cancer Res..

[39] Hu X., Fujimoto J., Ying L. (2019). Multi-region exome sequencing reveals genomic evolution from preneoplasia to lung adenocarcinoma. Nat. Commun..

[40] Xiang C., Ji C., Cai Y. (2022). Distinct mutational features across preinvasive and invasive subtypes identified through comprehensive profiling of surgically resected lung adenocarcinoma, Modern pathology : an. official journal of the United States and Canadian Academy of Pathology, Inc.

