# Supplementary material for: Targeted next-generation sequencing of 491 lung cancers in clinical practice: Implications for future detection strategy and targeted therapy
Source: Heliyon. 2024 Mar 7;10(6):e27591. doi: 10.1016/j.heliyon.2024.e27591 (PMC10944278; doi:10.1016/j.heliyon.2024.e27591)
Supplement: Multimedia component 2 [file mmc2.docx]

**Supplemental Table S2- List of *EGFR* targets of the ARMS assay**

| Exon | Protein change | Nucleic acid change | Cosmic ID |
| --- | --- | --- | --- |
| 18 | G719A | 2156G>C | 6239 |
|  | G719S | 2155G>A | 6252 |
|  | G719C | 2155G>T | 6253 |
| 19 | E746_A750del (1) | 2235_2249del l5 | 6223 |
|  | E746_A750del (2) | 2236_2250del l5 | 6225 |
|  | L747_P753>S | 2240_2257del l8 | 12370 |
|  | E746_T751>I | 2235_2252>AAT(complex) | 13551 |
|  | E746_T751del | 2236_2253del l8 | 12728 |
|  | E746_T751>A | 2237_2251del l5* | 12678 |
|  | E746_S752>A | 2237_2254del l8* | 12367 |
|  | E746_S752>V | 2237_2255>T(complex) | 12384 |
|  | E746_S752>D | 2238_2255del l8* | 6220 |
|  | L747_A750>P | 2238_2248>GC(complex)* | 12422 |
|  | L747_T751>Q | 2238_2252>GCA(complex) | 12419 |
|  | L747_E749del | 2239_2247delTTAAGAGAA | 6218 |
|  | L747_T751del | 2239_2253del l5* | / |
|  | L747_S752del | 2239_2256del l8 | 6255 |
|  | L747_A750>P | 2239_2248HAAGAGAAG>C(complex) | 12382 |
|  | L747_P753>Q | 2239_2258>CA(complex) | 12387 |
|  | L747_T751>S | 2240_2251del l2* | 6210 |
|  | L747_T751del | 2240_22Mdel l5 | 12369 |
|  | L747_T751>P | 2239_2251>C(complex) | 12383 |
|  | L747_T751del | 2238_2252del l5* | 23571 |
|  | L747_S752>Q | 2239_2256>CAA(complex)* | 12403 |
|  | E746_T751>V | 2237_2252>T(complex)* | 12386 |
|  | E746_T751>T | 2236_2253>ACG(complex)* | / |
|  | L747_A750>P | 2239_2250>CCC(complex)* | / |
|  | L747_K754>QL | 2239_2261>CAATT(complex)* | / |
|  | E746_K754>EQHL | 2238_2261>GCAACATCT(complex)* | / |
|  | L747_S752>Q | 2238_2256>GCAA(complex)* | 26441 |
|  | E746_A750>QP | 2236_2248*CAAC(complex)* | 13557 |
| 20 | T790M | 2369C>T | 6240 |
|  | H773_V774insH | 2319_2320insCAC* | 12377 |
|  | D770_N771insG | 2310_2311insGGT | 12378 |
|  | V769_D770insASV | 2307_2308insGCCAGCGTG* | 12376 |
|  | H773_V774insNPH | 2319_2320insAACCCCCAC* | 12381 |
|  | H773_V774insQ | 2319_2320insCAG* | 131552 |
|  | N771_P772insT | 2313_2314insACC* | / |
|  | N771_P772insH | 2314_2315insACC* | 1238031 |
|  | P772_H773insQ | 2318_2319insACA* | / |
|  | H773_V774insY | 2319_2320insTAC* | / |
|  | N771>GY | 2311A>GGTT | 53189 |
|  | D770_N771insG | 2310_2311insGGC* | 13004 |
|  | D770_N771insGD | 2308_2309insACGGCG* | 22955 |
|  | S768I | 2303G>T | 6241 |
| 21 | L858R | 2573T>G | 6224 |
|  | L861Q | 2582T>A | 6213 |
