# Supplementary material for: Targeted next-generation sequencing of 491 lung cancers in clinical practice: Implications for future detection strategy and targeted therapy
Source: Heliyon. 2024 Mar 7;10(6):e27591. doi: 10.1016/j.heliyon.2024.e27591 (PMC10944278; doi:10.1016/j.heliyon.2024.e27591)
Supplement: Multimedia component 3 [file mmc3.docx]

**Supplemental table 3** Discordant cases in mutations of EGFR detected by ARMS and targeted NGS

| **Num** | **Gender** | **Age** | **Diagnosis** | **sample type** | **ARMS-PCR** | **Targeted NGS** |
| --- | --- | --- | --- | --- | --- | --- |
| 301 | Male | 68 | SCLC | biopsied tissue | Ex19dels/S768I | E746_A750dels(E19) |
| 63 | Female | 62 | LUAD |  | L858R | L858R(E21); E709V(E18) |
| 432 | Female | 49 | LUAD | biopsied tissue | L858R | L858R(E21); R776H(E20) |
| 488 | Female | 80 | LUAD | Resected lung tissue | L858R | L858R(E21); K860I(E21); L792H(E20) |
| 184 | Male | 70 | LUAD | biopsied tissue | S768I | G724S(E18); S768I(E20) |
| 190 | Male | 59 | LUAD | biopsied tissue | Ex19dels |  |
| 356 | Male | 65 | LUAD | pleural effusion exfoliated cells | Ex19dels |  |
| 192 | Female | 62 | LUAD | biopsied tissue | Ex19dels/T790M |  |
| 16 | Male | 72 | LUAD |  | L858R |  |
| 384 | Male | 69 | LUAD | Resected lung tissue |  | E746_A750dels(E19) |
| 1 | Male | 49 | LUAD | pleural effusion exfoliated cells |  | L858R (E21); T790M (E20) |
| 73 | Female | 60 | LUAD | biopsied tissue |  | L858R (E21) |
| 2 | Female | 88 | LUAD | pleural effusion exfoliated cells |  | M793I (E20) |
| 5 | Male | 85 | LUAD | biopsied tissue |  | E709_T710dels insD(E18) |
| 8 | Male | 60 | LUAD | biopsied tissue |  | N771delsinsGF (E20) |
| 36 | Female | 67 | LUAD | biopsied tissue |  | I740_K745dup(E1 9) |
| 41 | Male | 66 | adenomas | Resected lung tissue |  | F712S(E18) |
| 48 | Female | 66 | LUAD | biopsied tissue |  | S752_I759dels (E19) |
| 56 | Female | 77 | LUAD | biopsied tissue |  | P772_H773dup (E20) |
| 77 | Male | 74 | LUAD | biopsied tissue |  | A871V(E21) |
| 114 | Male | 51 | LUSC | Resected lung tissue |  | I740_K745dup (E19) |
| 164 | Female | 63 | LUAD | biopsied tissue |  | P794S (E20) |
| 220 | Male | 59 | LUAD | biopsied tissue |  | G863S(E21) |
| 246 | Female | 69 | LUAD | Resected lung tissue |  | 772_H773insGA(E20) |
| 248 | Female | 58 | LUAD | Resected lung tissue |  | S752_I759dels(E19) |
| 256 | Female | 50 | LUAD | Resected lung tissue |  | L746_T753delsinsP(E19) |
| 266 | Male | 67 | LUAD | biopsied tissue |  | A859T(E21) |
| 276 | Female | 62 | LUAD | Resected lung tissue |  | H773delsinsQW(E20) |
| 291 | Male | 38 | LUAD | biopsied tissue |  | L751_T759delsinsP(E19） |
| 424 | Female | 69 | LUAD | pleural effusion exfoliated cells |  | E746K(E19) |
| 451 | Female | 59 | LUAD | Resected lung tissue |  | N771dup(E20) |
